# Supplementary material for: Cell cycle population effects in perturbation studies
Source: Mol Syst Biol. 2014 Jul 1;10(6):732. doi: 10.15252/msb.20145172 (PMC4265054; doi:10.15252/msb.20145172)
Supplement: Supplementary file 1 — Supplementary Figure S1 [file msb0010-0732-sd1.pdf]

Supplementary Figure 1

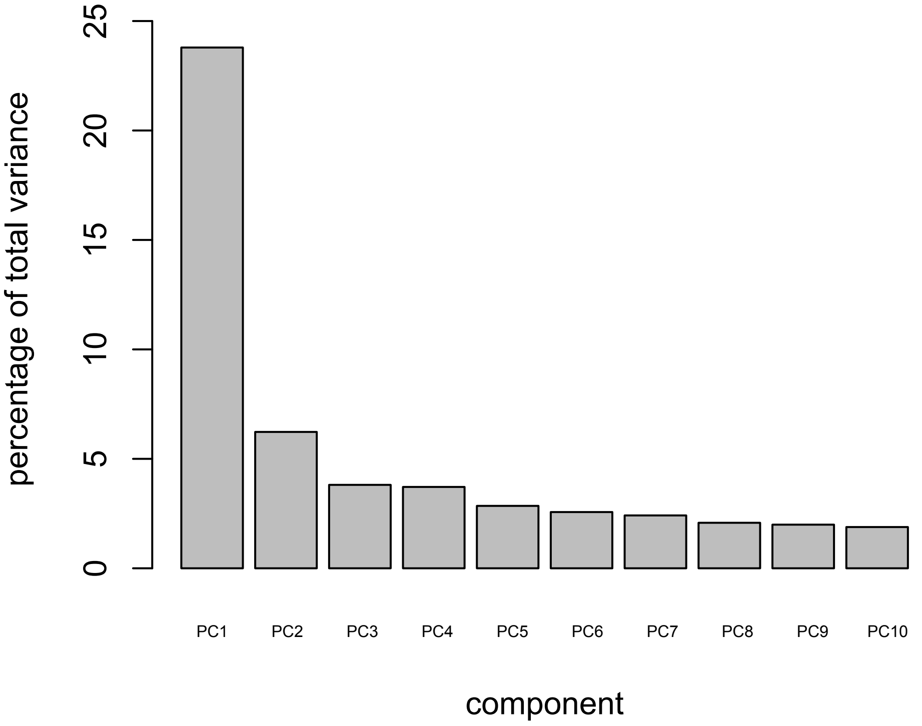

Supplementary figure S1: Principal component analysis.
The percentage of variance captured by the first 10 principal components. The slow growth profile corresponds to the first principal component and represents 24% of the total variance.
