# Supplementary material for: Cell cycle population effects in perturbation studies
Source: Mol Syst Biol. 2014 Jul 1;10(6):732. doi: 10.15252/msb.20145172 (PMC4265054; doi:10.15252/msb.20145172)
Supplement: Supplementary file 2 — Supplementary Figure S2 [file msb0010-0732-sd2.pdf]

Supplementary Figure 2

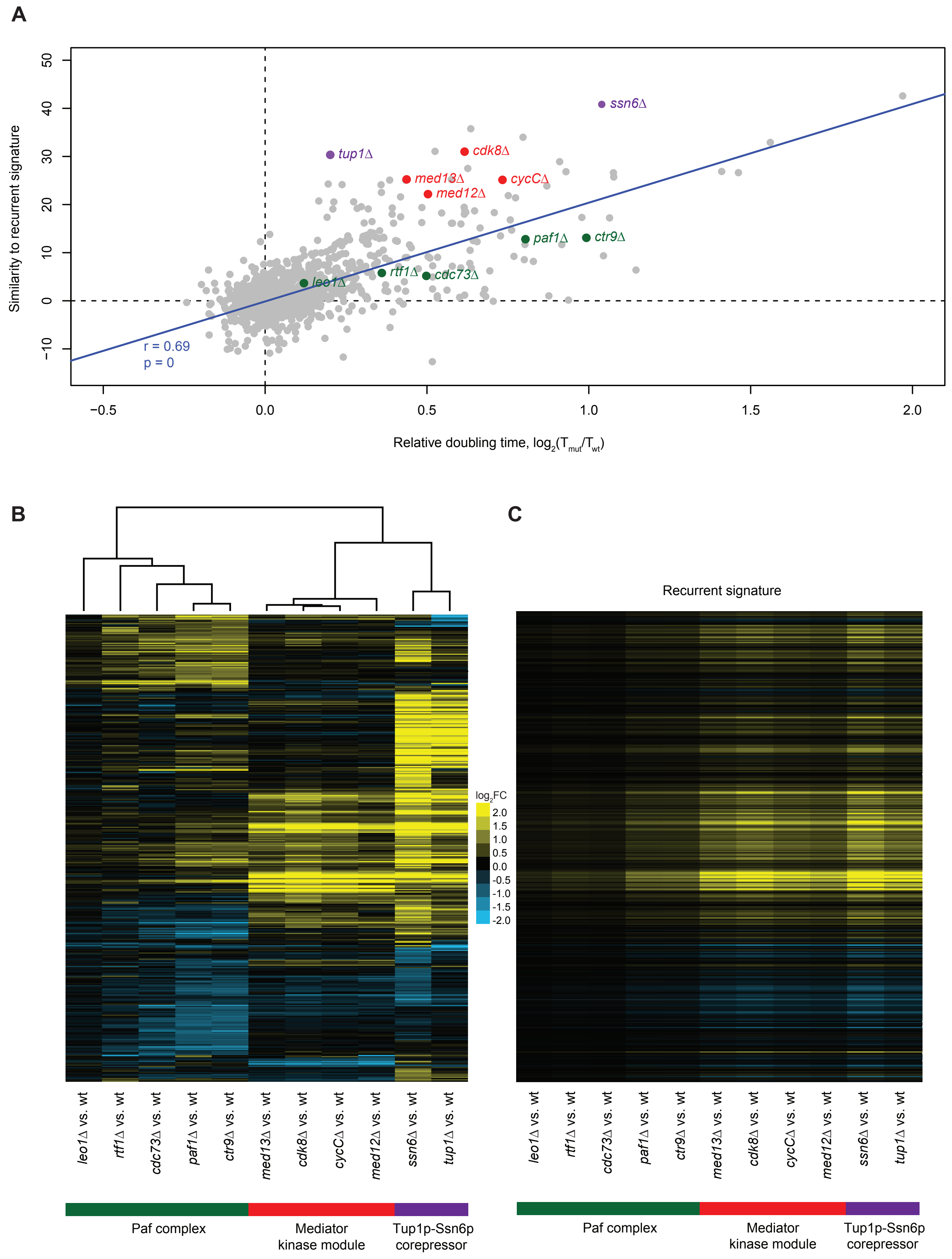

Supplementary Figure S2. Examples of specific gene expression changes associated with off-diagonal deletion strains from Figure 1

(**A**) The similarities of deletion profiles to the recurrent signature, versus their growth-rate plotted as log2 (doubling time in mutant/doubling time wild type). The similarity is expressed as the projection of a deletion profile onto the normalized recurrent profile (this is proportional to their covariance). Highlighted are 11 off-diagonal deletion mutants that make up the Paf-complex (green), mediator kinase submodule (red) and the Tup1p-Cyc8p corepressor complex (purple). (**B**) Heat map of mRNA expression changes (y-axis) more than 1.7-fold and with $p<0.05$ compared to wild type in the 11 deletion mutants (x-axis) highlighted in (A). Both the transcripts and deletion strains have been clustered (cosine correlation; average linkage). (**C**) Heatmap of the recurrent signature of the 11 deletion mutants shown in (B). Transcripts and deletions strains are ordered as in (B).
