# Supplementary material for: Cell cycle population effects in perturbation studies
Source: Mol Syst Biol. 2014 Jul 1;10(6):732. doi: 10.15252/msb.20145172 (PMC4265054; doi:10.15252/msb.20145172)
Supplement: Supplementary file 3 — Supplementary Figure S3 [file msb0010-0732-sd3.pdf]

Supplementary figure 3

A

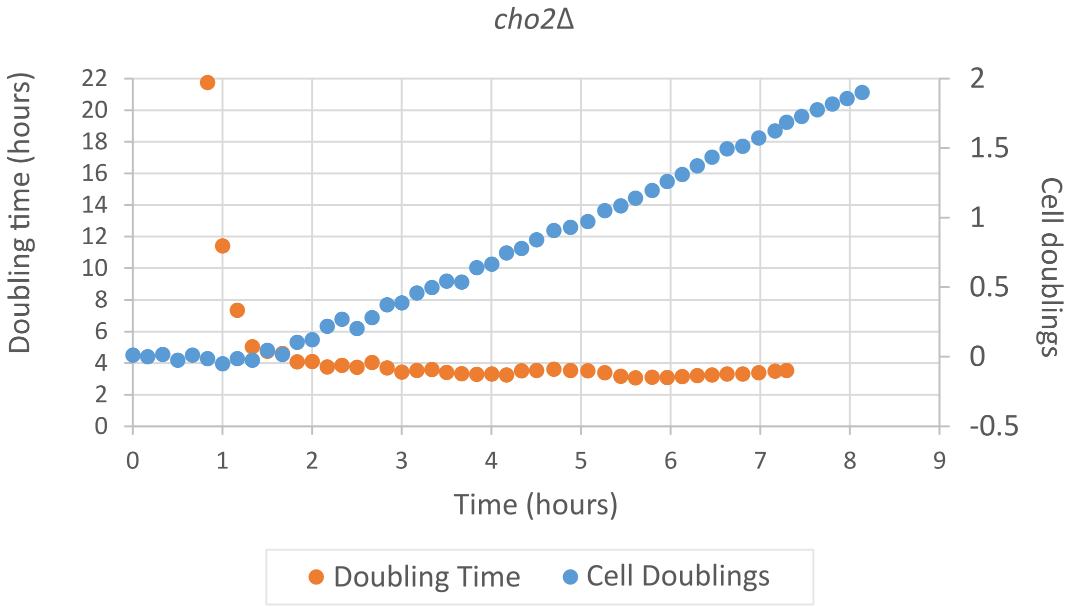

B

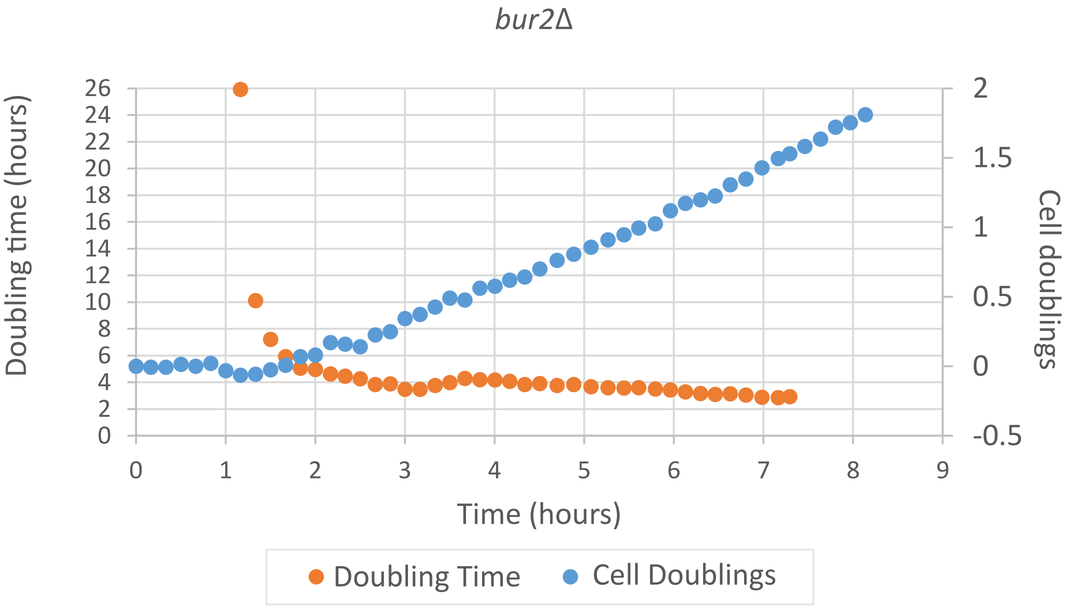

C

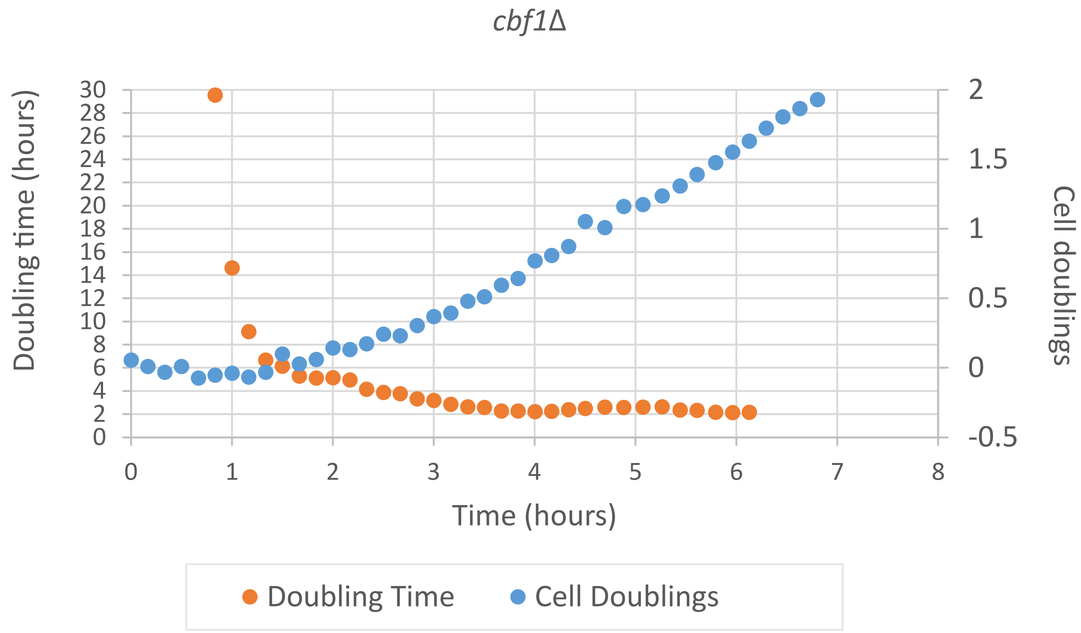

D

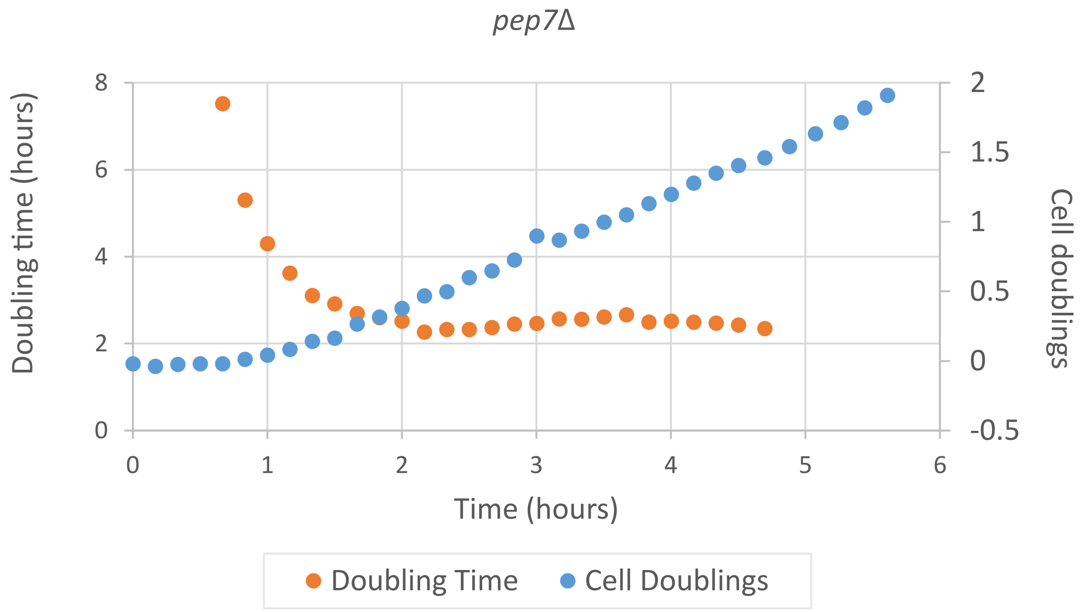

Supplementary Figure S3. Deletion mutants have attained balanced growth. (**A-D**) Plots showing the doubling time (orange dots, left y-axis) and cell doublings (blue dots, right y-axis) over time of the four slowest deletion strains in figure 5. Doubling times were calculated over a sliding window containing 10 optical density measurements.
