# Supplementary material for: Cell cycle population effects in perturbation studies
Source: Mol Syst Biol. 2014 Jul 1;10(6):732. doi: 10.15252/msb.20145172 (PMC4265054; doi:10.15252/msb.20145172)
Supplement: Supplementary file 4 — Supplementary Figure S4 [file msb0010-0732-sd4.pdf]

Supplementary figure 4

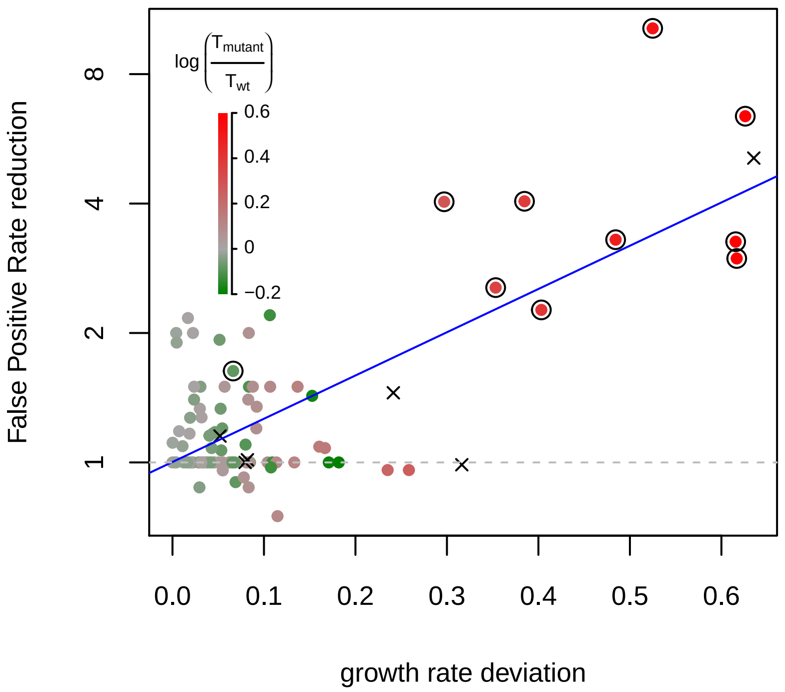

Supplementary figure S4: Relationship between false positive rate reduction and growth rate. The False Positive Rate (FPR) reduction achieved by using the transformed data increases for greater deviations from wild type growth rate. The growth rate deviation is expressed as the absolute value of the relative doubling time; the FPR reduction is shown in log2-scale, and is relative to the FPR in the original data. Data points are coloured by their relative doubling time, as in Figure 6C. Crosses are data points of transcription factors for which the FPR is greater than 0.01 (both in the original and the transformed data). Circled points are transcription factors whose FPR has improved to better than 0.01 as a result of the transformation. The correlation of the linear regression (blue line) is 0.74
