# Supplementary material for: Cell cycle population effects in perturbation studies
Source: Mol Syst Biol. 2014 Jul 1;10(6):732. doi: 10.15252/msb.20145172 (PMC4265054; doi:10.15252/msb.20145172)
Supplement: Supplementary file 5 — Supplementary Figure S5 [file msb0010-0732-sd5.pdf]

Supplementary figure 5

A

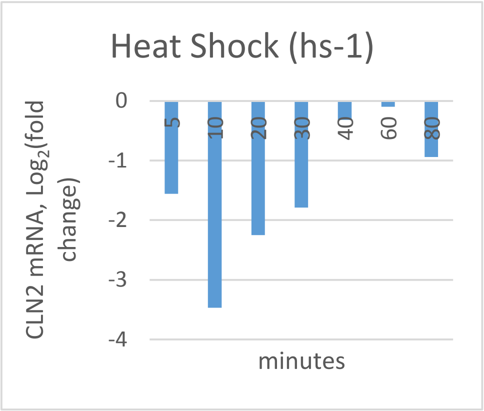

B

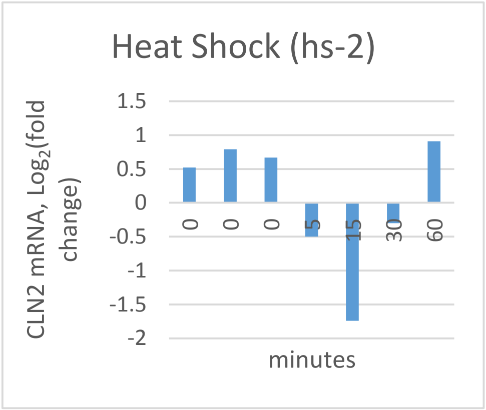

C

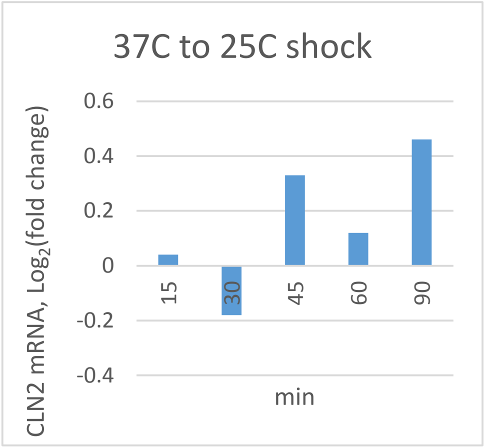

D

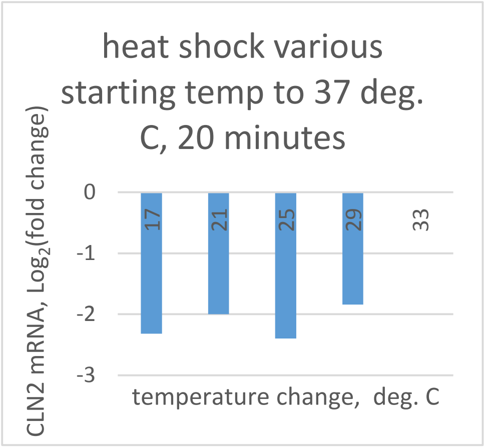

E

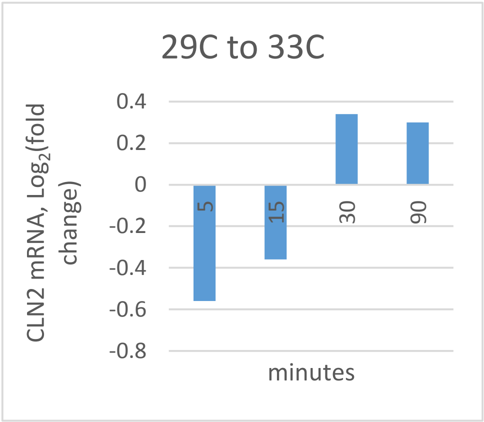

F

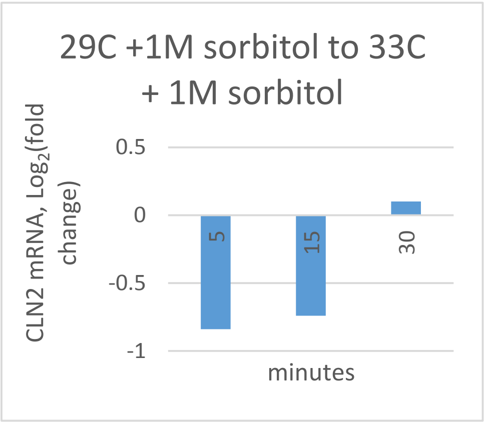

G

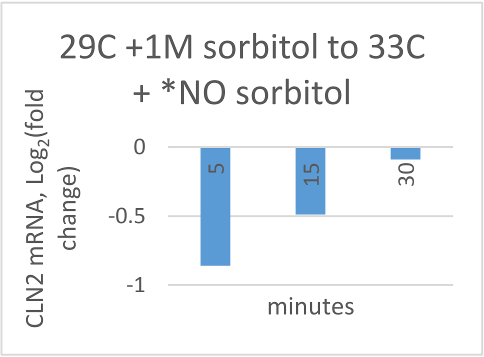

H

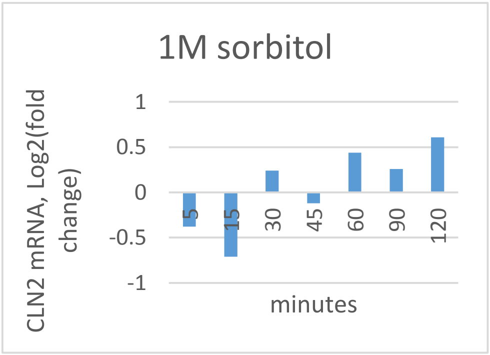

I

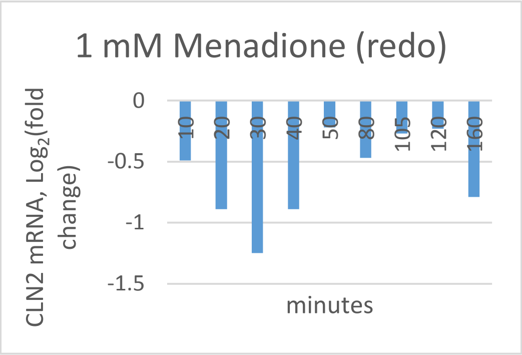
1 mM Menadione (redo)
CLN2 mRNA, Log2(fold change)
0
-0.5
-1
-1.5
10 20 30 40 50 80 105 120 160
minutes

J

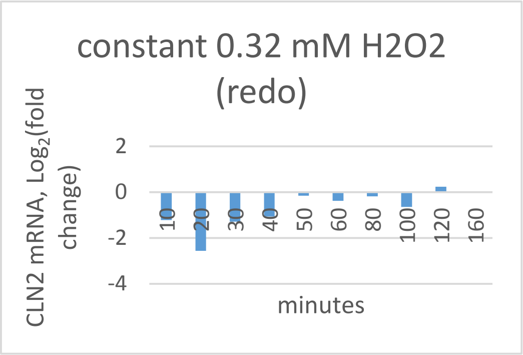
constant 0.32 mM H2O2 (redo)
CLN2 mRNA, Log2(fold change)
2
0
-2
-4
10 20 30 40 50 60 80 100 120 160
minutes

K

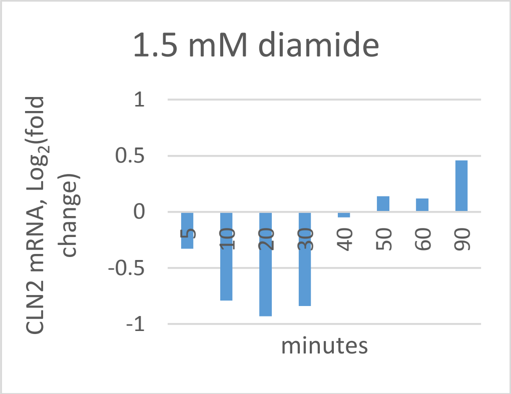
1.5 mM diamide
CLN2 mRNA, Log2(fold change)
1
0.5
0
-0.5
-1
5 10 20 30 40 50 60 90
minutes

L

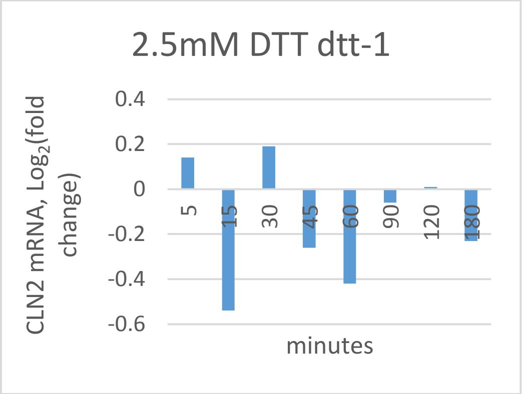
2.5mM DTT dtt-1
CLN2 mRNA, Log2(fold change)
0.4
0.2
0
-0.2
-0.4
-0.6
5 15 30 45 60 90 120 180
minutes

M

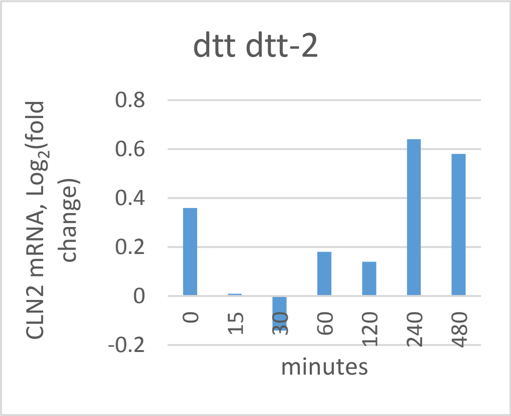
dtt dtt-2
CLN2 mRNA, Log2(fold change)
0.8
0.6
0.4
0.2
0
-0.2
0 15 30 60 120 240 480
minutes

N

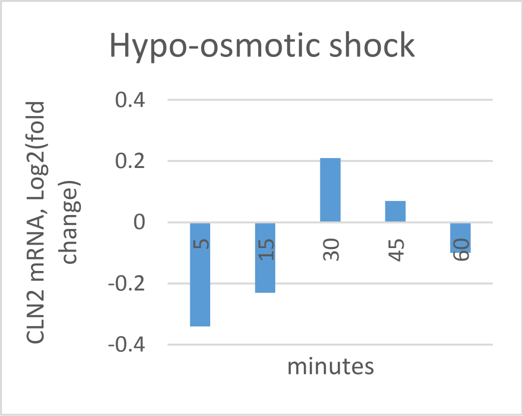
Hypo-osmotic shock
CLN2 mRNA, Log2(fold change)
0.4
0.2
0
-0.2
-0.4
5 15 30 45 60
minutes

O

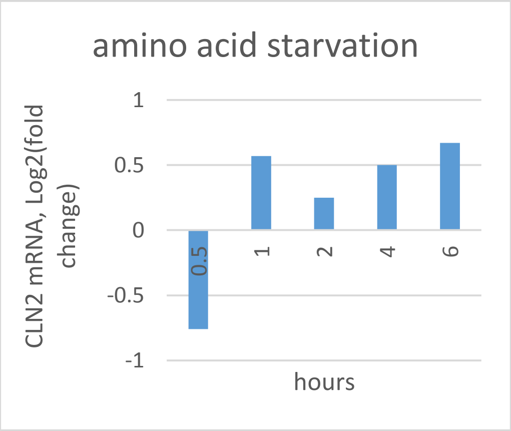
amino acid starvation
CLN2 mRNA, Log2(fold change)
1
0.5
0
-0.5
-1
0.5 1 2 4 6
hours

P

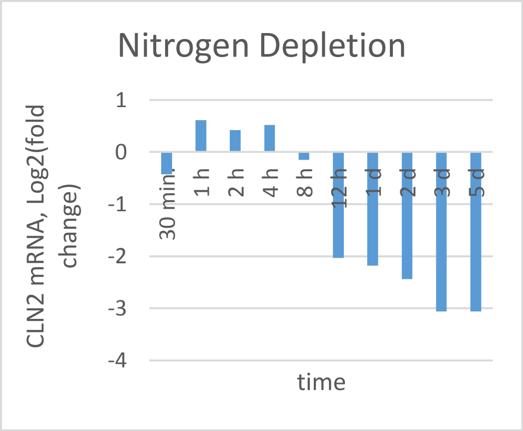
Nitrogen Depletion
CLN2 mRNA, Log2(fold change)
1
0
-1
-2
-3
-4
30 min. 1 h 2 h 4 h 8 h 12 h 1 d 2 d 3 d 5 d
time

Q

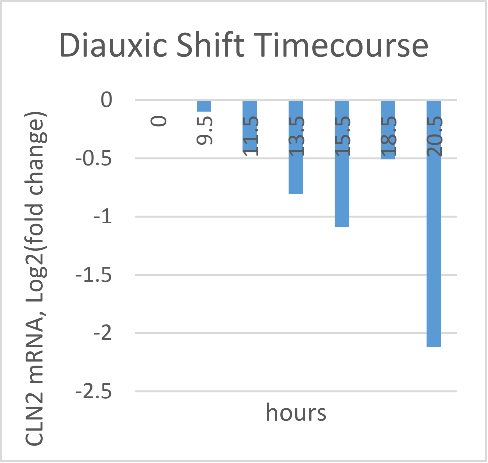

R

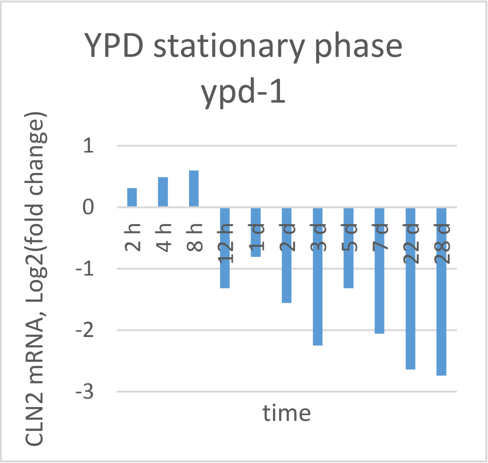

S

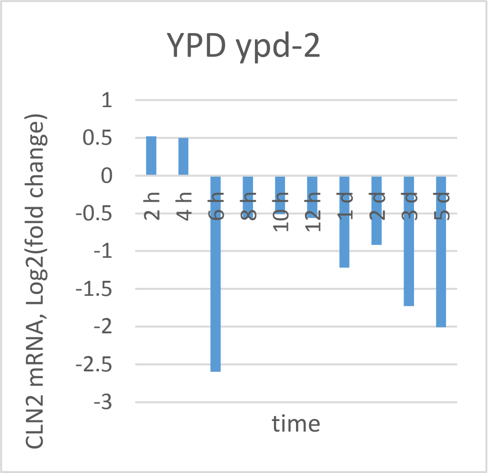

Supplementary figure S5. CLN2 mRNA expression during stress time courses from Gasch et. al., study. (**A-S**) CLN2 mRNA expression.
