# Supplementary material for: Cell cycle population effects in perturbation studies
Source: Mol Syst Biol. 2014 Jul 1;10(6):732. doi: 10.15252/msb.20145172 (PMC4265054; doi:10.15252/msb.20145172)
Supplement: Supplementary file 6 — Supplementary Figure S6 [file msb0010-0732-sd6.pdf]

Supplementary figure 6

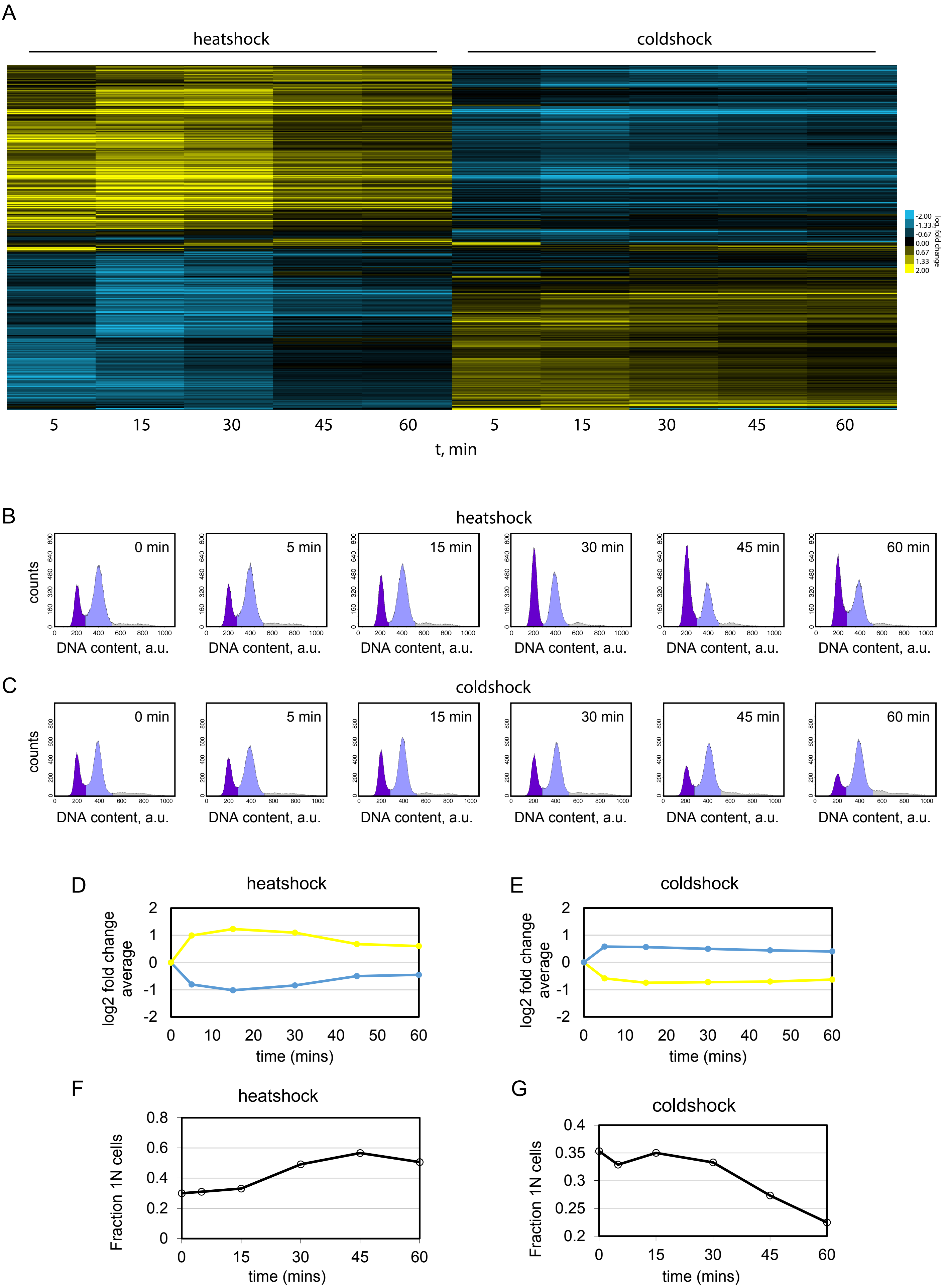

Supplementary figure S6. Cold shifted cells display the opposite flow cytometry profile shift compared to heat shocked cells. (**A**) Heat map of mRNA expression changes during a heat shock and cold shock time series including all transcripts changing more than 1.7-fold and $p<0.05$ in any single time point compared to t=0. (**B**) Time series of flow cytometry profiles after heat shock. (**C**) Time series of flow cytometry profiles after cold shock. (**D**) Average magnitude of gene expression changes as shown in (A) above for genes with increased (yellow) or decreased (blue) expression during heat shock. (**E**) Average magnitude of gene expression changes during cold shock, as shown in (A) above, colours as in (D). (**F**) Fraction of 1N cells during heat shock. (**G**) Fraction of 1N cells during cold shock.
