# Supplementary material for: Cell cycle population effects in perturbation studies
Source: Mol Syst Biol. 2014 Jul 1;10(6):732. doi: 10.15252/msb.20145172 (PMC4265054; doi:10.15252/msb.20145172)
Supplement: Supplementary file 7 — Supplementary Table S1 [file msb0010-0732-sd7.pdf]

SUPPLEMENTARY TABLE S1: GO and GSTF binding enrichments for transcripts changing in the slow growth signature

The slow growth signature with gene expression changes corresponding to the slowest growing strain was used for enrichment analysis. Transcripts were used based on a fold-change cutoff of 1.7 and a p-value cutoff of 0.05, resulting in 439 transcripts with increased expression and 207 transcripts with decreased expression. GO terms and GSTF enrichments were considered significant with a p-value less than 0.01 (after Bonferroni correction). Terms also found in the ESR have been marked by an asterix (*). P-value = 0 are below machine precision.

Transcripts with increased expression:

Gene Ontology - Biological Process	Corrected p-value	Cluster frequency	Background frequency
oxidation-reduction process (GO:0055114)*	0.00	95/426 (22.3%)	429/6168 (6.96%)
generation of precursor metabolites and energy (GO:0006091)	0.00	51/426 (12%)	171/6168 (2.77%)
energy derivation by oxidation of organic compounds (GO:0015980)	0.00	44/426 (10.3%)	145/6168 (2.35%)
oxidative phosphorylation (GO:0006119)	0.00	17/426 (3.99%)	29/6168 (0.47%)
cellular carbohydrate metabolic process (GO:0044262)*	0.00	39/426 (9.15%)	154/6168 (2.5%)
cellular respiration (GO:0045333)	0.00	31/426 (7.28%)	102/6168 (1.65%)
carbohydrate metabolic process (GO:0005975)*	2.07E-09	57/426 (13.4%)	309/6168 (5.01%)
ATP synthesis coupled electron transport (GO:0042773)	0.00	16/426 (3.76%)	28/6168 (0.454%)
mitochondrial ATP synthesis coupled electron transport (GO:0042775)	0.00	16/426 (3.76%)	28/6168 (0.454%)
respiratory electron transport chain (GO:0022904)	0.00	16/426 (3.76%)	30/6168 (0.486%)
single-organism carbohydrate catabolic process (GO:0044724)*	0.00	26/426 (6.1%)	83/6168 (1.35%)
electron transport chain (GO:0022900)	0.00	16/426 (3.76%)	31/6168 (0.503%)
single-organism carbohydrate metabolic process (GO:0044723)	0.00	50/426 (11.7%)	266/6168 (4.31%)
aerobic respiration (GO:0009060)	3.21E-08	26/426 (6.1%)	85/6168 (1.38%)
carbohydrate catabolic process (GO:0016052)*	0.00	26/426 (6.1%)	90/6168 (1.46%)
trehalose metabolic process (GO:0005991)*	1.47E-06	9/426 (2.11%)	11/6168 (0.178%)
biological_process (GO:0008150)*	0.00	109/426 (25.6%)	922/6168 (14.9%)
cellular response to oxidative stress (GO:0034599)*	0.00	25/426 (5.87%)	98/6168 (1.59%)
response to oxidative stress (GO:0006979)*	0.00	25/426 (5.87%)	104/6168 (1.69%)
single-organism catabolic process (GO:0044712)*	2.09E-05	58/426 (13.6%)	399/6168 (6.47%)
single-organism metabolic process (GO:0044710)*	0.00	182/426 (42.7%)	1900/6168 (30.8%)
mitochondrial electron transport, ubiquinol to cytochrome c (GO:0006122)	0.00	8/426 (1.88%)	11/6168 (0.178%)
monosaccharide metabolic process (GO:0005996)	0.00	20/426 (4.69%)	79/6168 (1.28%)
phosphorus metabolic process (GO:0006793)	0.00	83/426 (19.5%)	705/6168 (11.4%)
glycogen biosynthetic process (GO:0005978)*	0.00	8/426 (1.88%)	13/6168 (0.211%)
phosphate-containing compound metabolic process (GO:0006796)	0.0005545	80/426 (18.8%)	680/6168 (11%)
vacuolar protein catabolic process (GO:0007039)	0.00	7/426 (1.64%)	10/6168 (0.162%)
pyridine nucleotide metabolic process (GO:0019362)*	0.0008791	15/426 (3.52%)	51/6168 (0.827%)
cellular response to chemical stimulus (GO:0070887)*	0.00	44/426 (10.3%)	300/6168 (4.86%)
mitochondrial electron transport, succinate to ubiquinone (GO:0006121)	0.001405	5/426 (1.17%)	5/6168 (0.0811%)
phosphorylation (GO:0016310)	0.001451	40/426 (9.39%)	265/6168 (4.3%)
tricarboxylic acid cycle (GO:0006099)	0.00	11/426 (2.58%)	29/6168 (0.47%)
response to abiotic stimulus (GO:0009628)	0.00	28/426 (6.57%)	157/6168 (2.55%)
energy reserve metabolic process (GO:0006112)	0.00	11/426 (2.58%)	30/6168 (0.486%)
cellular carbohydrate biosynthetic process (GO:0034637)	0.00	14/426 (3.29%)	48/6168 (0.778%)
organic substance catabolic process (GO:1901575)*	0.002322	78/426 (18.3%)	681/6168 (11%)
cellular carbohydrate catabolic process (GO:0044275)	0.003889	14/426 (3.29%)	50/6168 (0.811%)
nicotinamide nucleotide metabolic process (GO:0046496)	0.003889	14/426 (3.29%)	50/6168 (0.811%)
pyridine-containing compound metabolic process (GO:0072524)	0.004129	16/426 (3.76%)	64/6168 (1.04%)

catabolic process (GO:0009056)*	0.005871	85/426 (20%)	780/6168 (12.6%)
pentose metabolic process (GO:0019321)*	0.007776	7/426 (1.64%)	13/6168 (0.211%)
glycogen metabolic process (GO:0005977)	0.00861	10/426 (2.35%)	28/6168 (0.454%)
disaccharide metabolic process (GO:0005984)	0.00861	10/426 (2.35%)	28/6168 (0.454%)
monosaccharide catabolic process (GO:0046365)	0.009165	11/426 (2.58%)	34/6168 (0.551%)

**Gene Specific Transcription Factor**	**Corrected p-value**	**Cluster frequency**	**Background frequency**
MSN4*	1.82E-10	49/440 (11.1%)	161/6168 (2.61%)
MSN2*	1.87E-10	54/440 (12.3%)	182/6168 (2.95%)
SKN7	5.37E-10	44/440 (10%)	195/6168 (3.16%)
HAP4	4.33E-08	20/440 (4.55%)	55/6168 (0.891%)
HSF1	1.94E-05	15/440 (3.41%)	44/6168 (0.713%)
NRG1	0.0003599	28/440 (6.36%)	154/6168 (2.5%)
ROX1	0.001636	20/440 (4.55%)	98/6168 (1.59%)
UME6	0.002122	31/440 (7.05%)	196/6168 (3.18%)
XBP1	0.006913	14/440 (3.18%)	60/6168 (0.972%)

Transcripts with decreased expression:

**Gene Ontology - Biological Process**	**Corrected p-value**	**Cluster frequency**	**Background frequency**
ribosome biogenesis (GO:0042254)*	8.82E-70	98/202 (48.5%)	346/6168 (5.61%)
ribonucleoprotein complex biogenesis (GO:0022613)*	8.02E-61	98/202 (48.5%)	420/6168 (6.81%)
rRNA processing (GO:0006364)*	2.15E-54	76/202 (37.6%)	249/6168 (4.04%)
rRNA metabolic process (GO:0016072)*	5.69E-52	76/202 (37.6%)	266/6168 (4.31%)
ncRNA processing (GO:0034470)*	2.24E-47	81/202 (40.1%)	356/6168 (5.77%)
ncRNA metabolic process (GO:0034660)*	4.41E-44	83/202 (41.1%)	414/6168 (6.71%)
ribosomal large subunit biogenesis (GO:0042273)*	2.40E-39	44/202 (21.8%)	93/6168 (1.51%)
RNA processing (GO:0006396)*	1.11E-35	84/202 (41.6%)	539/6168 (8.74%)
cellular component biogenesis (GO:0044085)*	6.97E-29	100/202 (49.5%)	948/6168 (15.4%)
ribosomal small subunit biogenesis (GO:0042274)*	1.51E-22	36/202 (17.8%)	125/6168 (2.03%)
RNA metabolic process (GO:0016070)*	1.33E-20	97/202 (48%)	1123/6168 (18.2%)
maturation of SSU-rRNA from tricistronic rRNA transcript (SSU-rRNA, 5.8S rRNA, LSU-rRN	7.13E-20	30/202 (14.9%)	94/6168 (1.52%)
maturation of SSU-rRNA (GO:0030490)*	1.44E-18	30/202 (14.9%)	103/6168 (1.67%)
maturation of 5.8S rRNA (GO:0000460)*	2.99E-18	27/202 (13.4%)	81/6168 (1.31%)
maturation of 5.8S rRNA from tricistronic rRNA transcript (SSU-rRNA, 5.8S rRNA, LSU-rRNA) (GO:0000466)	2.99E-18	27/202 (13.4%)	81/6168 (1.31%)
maturation of LSU-rRNA from tricistronic rRNA transcript (SSU-rRNA, 5.8S rRNA, LSU-rRNA) (GO:0000463)*	1.27E-16	18/202 (8.91%)	32/6168 (0.519%)
maturation of LSU-rRNA (GO:0000470)*	4.14E-15	18/202 (8.91%)	37/6168 (0.6%)
nucleobase-containing compound metabolic process (GO:0006139)*	4.88E-15	112/202 (55.4%)	1703/6168 (27.6%)
cellular nitrogen compound metabolic process (GO:0034641)*	7.76E-15	117/202 (57.9%)	1845/6168 (29.9%)
heterocycle metabolic process (GO:0046483)*	1.00E-14	115/202 (56.9%)	1797/6168 (29.1%)
organic cyclic compound metabolic process (GO:1901360)*	3.51E-14	116/202 (57.4%)	1851/6168 (30%)
endonucleolytic cleavage involved in rRNA processing (GO:0000478)*	3.74E-14	19/202 (9.41%)	47/6168 (0.762%)
endonucleolytic cleavage of tricistronic rRNA transcript (SSU-rRNA, 5.8S rRNA, LSU-rRNA) (GO:0000479)*	3.74E-14	19/202 (9.41%)	47/6168 (0.762%)
cellular aromatic compound metabolic process (GO:0006725)*	5.84E-14	113/202 (55.9%)	1782/6168 (28.9%)
RNA phosphodiester bond hydrolysis, endonucleolytic (GO:0090502)*	6.02E-14	19/202 (9.41%)	48/6168 (0.778%)
nitrogen compound metabolic process (GO:0006807)*	7.57E-14	122/202 (60.4%)	2033/6168 (33%)

endonucleolytic cleavage in ITS1 to separate SSU-rRNA from 5.8S rRNA and LSU-rRNA from tricistronic rRNA transcript (SSU-rRNA, 5.8S rRNA, LSU-rRNA) (GO:0000447)*	1.20E-13	18/202 (8.91%)	43/6168 (0.697%)
nucleic acid metabolic process (GO:0090304)*	6.06E-13	98/202 (48.5%)	1448/6168 (23.5%)
cleavage involved in rRNA processing (GO:0000469)*	7.12E-13	21/202 (10.4%)	69/6168 (1.12%)
endonucleolytic cleavage in 5'-ETS of tricistronic rRNA transcript (SSU-rRNA, 5.8S rRNA, LSU-rRNA) (GO:0000480)*	7.80E-13	15/202 (7.43%)	29/6168 (0.47%)
RNA phosphodiester bond hydrolysis (GO:0090501)*	9.89E-13	21/202 (10.4%)	70/6168 (1.13%)
endonucleolytic cleavage to generate mature 5'-end of SSU-rRNA from (SSU-rRNA, 5.8S rRNA, LSU-rRNA) (GO:0000472)*	5.22E-12	15/202 (7.43%)	32/6168 (0.519%)
rRNA 5'-end processing (GO:0000967)*	9.31E-12	15/202 (7.43%)	33/6168 (0.535%)
ncRNA 5'-end processing (GO:0034471)*	9.31E-12	15/202 (7.43%)	33/6168 (0.535%)
gene expression (GO:0010467)*	1.54E-11	103/202 (51%)	1645/6168 (26.7%)
RNA 5'-end processing (GO:0000966)*	1.62E-11	15/202 (7.43%)	34/6168 (0.551%)
ribosome assembly (GO:0042255)*	5.79E-11	18/202 (8.91%)	58/6168 (0.94%)
ribosomal large subunit assembly (GO:0000027)*	3.90E-10	14/202 (6.93%)	34/6168 (0.551%)
ribosomal subunit export from nucleus (GO:0000054)*	4.52E-09	15/202 (7.43%)	47/6168 (0.762%)
ribosome localization (GO:0033750)*	4.52E-09	15/202 (7.43%)	47/6168 (0.762%)
establishment of ribosome localization (GO:0033753)*	4.52E-09	15/202 (7.43%)	47/6168 (0.762%)
ribonucleoprotein complex localization (GO:0071166)*	4.52E-09	15/202 (7.43%)	47/6168 (0.762%)
ribonucleoprotein complex export from nucleus (GO:0071426)*	4.52E-09	15/202 (7.43%)	47/6168 (0.762%)
rRNA-containing ribonucleoprotein complex export from nucleus (GO:0071428)*	4.52E-09	15/202 (7.43%)	47/6168 (0.762%)
ribonucleoprotein complex assembly (GO:0022618)*	6.56E-08	23/202 (11.4%)	143/6168 (2.32%)
ribonucleoprotein complex subunit organization (GO:0071826)*	2.03E-07	23/202 (11.4%)	151/6168 (2.45%)
nucleic acid phosphodiester bond hydrolysis (GO:0090305)*	5.10E-07	25/202 (12.4%)	186/6168 (3.02%)
organelle assembly (GO:0070925)*	1.61E-06	19/202 (9.41%)	114/6168 (1.85%)
rRNA modification (GO:0000154)*	1.00E-05	9/202 (4.46%)	23/6168 (0.373%)
RNA methylation (GO:0001510)*	1.32E-05	11/202 (5.45%)	39/6168 (0.632%)
cellular component organization or biogenesis (GO:0071840)*	1.84E-05	105/202 (52%)	2095/6168 (34%)
ribosomal large subunit export from nucleus (GO:0000055)*	5.14E-05	9/202 (4.46%)	27/6168 (0.438%)
nucleocytoplasmic transport (GO:0006913)*	9.40E-05	20/202 (9.9%)	160/6168 (2.59%)
nuclear transport (GO:0051169)*	0.0001043	20/202 (9.9%)	161/6168 (2.61%)
methylation (GO:0032259)*	0.000381	15/202 (7.43%)	101/6168 (1.64%)
macromolecule methylation (GO:0043414)*	0.0004119	13/202 (6.44%)	76/6168 (1.23%)
establishment of organelle localization (GO:0051656)*	0.0004341	15/202 (7.43%)	102/6168 (1.65%)
nuclear export (GO:0051168)*	0.0008006	16/202 (7.92%)	121/6168 (1.96%)
rRNA methylation (GO:0031167)	0.0008405	6/202 (2.97%)	13/6168 (0.211%)
primary metabolic process (GO:0044238)*	0.0008969	136/202 (67.3%)	3193/6168 (51.8%)
assembly of large subunit precursor of preribosome (GO:1902626)*	0.0009688	5/202 (2.48%)	8/6168 (0.13%)
cellular metabolic process (GO:0044237)*	0.0009805	140/202 (69.3%)	3327/6168 (53.9%)
cellular macromolecule metabolic process (GO:0044260)*	0.001081	110/202 (54.5%)	2398/6168 (38.9%)
organic substance metabolic process (GO:0071704)*	0.00184	139/202 (68.8%)	3323/6168 (53.9%)
macromolecule metabolic process (GO:0043170)*	0.002667	112/202 (55.4%)	2497/6168 (40.5%)
RNA modification (GO:0009451)*	0.003005	14/202 (6.93%)	104/6168 (1.69%)
cellular process (GO:0009987)*	0.003692	171/202 (84.7%)	4471/6168 (72.5%)
metabolic process (GO:0008152)*	0.006524	145/202 (71.8%)	3581/6168 (58.1%)

Gene Specific Transcription Factor

No significant enrichments found
