# Supplementary material for: Cell cycle population effects in perturbation studies
Source: Mol Syst Biol. 2014 Jul 1;10(6):732. doi: 10.15252/msb.20145172 (PMC4265054; doi:10.15252/msb.20145172)
Supplement: Supplementary file 9 — Description of Supplementary Data [file msb0010-0732-sd9.pdf]

Description of Supplementary data files

Supplementary_data1.txt - The first SVD mode of the gene expression changes of 1484 *S. cerevisiae* gene deletion mutants. This data is shown in Figure 1B, and is the original data (Figure 1A) projected onto the first principal component axis. Rows are transcripts, named by their systematic name, columns are gene deletion mutants, named by the common name of the gene that was deleted. Values are M values, that is, the log2(deletion mutant / wild type).

Supplementary_data2.txt - Relative growth rates of gene deletion strains, and the similarity of their gene expression to the slow growth signature (expressed as their covariance).

Supplementary_data3.txt - Original, untransformed gene expression changes of 1484 *S. cerevisiae* gene deletion mutants. Rows and column labels and ordering as in Supplementary_data1.txt

Supplementary_data4.txt - The slow growth profile. This is the column in the first SVD mode (see Figure 1B and Supplementary_data1) having the largest Euclidean norm. It corresponds to the scores of the first principal component scaled to that of the strain with the strongest slow growth effect. It can be thought of as the most severe slow growth related log2(fold change), per transcript, seen in the set of deletion mutants.

Supplementary_data5.txt - Transformed gene expression changes of 1484 *S. cerevisiae* gene deletion mutants. Any similarity to the slow growth profile has been removed by subtracting the first SVD mode from the original data. Rows, column labels and ordering as in Supplementary_data1.txt

Supplementary_data6.txt - Gene expression changes (as Supplementary_data3.txt), transformed gene expression changes (as Supplementary_data5.txt) and p-values of 1484 S. *cerevisiae* gene deletion mutants combined into one file. The p-values are those from Kemmeren et al. 2014, reflecting the significance of the untransformed expression changes.

Supplementary_movie1.mpg - Side by side animation of simulated cell cycle populations and experimental expression changes after heat shock. On the left, a simulated time course of the distribution of cell numbers over the phases of the cell cycle after heat shock is shown. The area of each wedge is proportional to the relative number of cells in the cycle phases indicated by the 'dial'. The red line halfway G1 represents the START check point. Purple indicates cells with 1N DNA content, blue those with 2N DNA content. On the right, a time course of expression changes after heat shock is shown. The experimental time points were taken at t=0, 5, 15, 30, 45 and 60 minutes, and interpolated linearly.

s3c2_1.1.1.zip - R package for the simulation, windows version

s3c2_1.1.1.tar.gz - R package for the simulation, Linux/Mac version

dccd_1.1.1.zip - R package for the deconvolution, windows version

dccd_1.1.1.tar.gz - R package for the deconvolution, Linux/Mac version

remove.signature.R -Script transforming any data using the slow growth profile determined in this study

svd-transform.R - Script that determines and subtracts the first principal component from any data set
