# Supplementary material for: Cell cycle population effects in perturbation studies
Source: Mol Syst Biol. 2014 Jul 1;10(6):732. doi: 10.15252/msb.20145172 (PMC4265054; doi:10.15252/msb.20145172)
Supplement: Supplementary file 16 — Supplementary R Packages [file msb0010-0732-sd16.zip › code/Rpackages/s3c2_1.1.1.zip!/s3c2/html/00Index.html]

R: Simple simulation of stressed cell counts.

# Simple simulation of stressed cell counts.

---

## Documentation for package ‘s3c2’ version 1.1.1

- DESCRIPTION file.

## Help Pages

|  |  |
| --- | --- |
| simulation | Simple simulation of stressed cell counts, implements the procedure used in O'Duibhir et al., Mol. Sys. Biol. 2014. |
