# Supplementary material for: Cell cycle population effects in perturbation studies
Source: Mol Syst Biol. 2014 Jul 1;10(6):732. doi: 10.15252/msb.20145172 (PMC4265054; doi:10.15252/msb.20145172)
Supplement: Supplementary file 16 — Supplementary R Packages [file msb0010-0732-sd16.zip › code/Rpackages/dccd_1.1.1.tar.gz!/dccd/test/win-builder.r-project.org/875Y9QCZF547/dccd_1.1.1.zip!/dccd/html/00Index.html]

R: Deconvolution using Cell Cycle Data

# Deconvolution using Cell Cycle Data

---

## Documentation for package ‘dccd’ version 1.1.1

- DESCRIPTION file.

## Help Pages

|  |  |
| --- | --- |
| deconvolve | Deconvolve using Cell Cycle Data, implements the procedure described in O'Duibhir et al., Mol. Sys. Biol. 2014. |
