# Supplementary material for: Cell cycle population effects in perturbation studies
Source: Mol Syst Biol. 2014 Jul 1;10(6):732. doi: 10.15252/msb.20145172 (PMC4265054; doi:10.15252/msb.20145172)
Supplement: Supplementary file 16 — Supplementary R Packages [file msb0010-0732-sd16.zip › code/Rpackages/dccd_1.1.1.tar.gz!/dccd/test/win-builder.r-project.org/875Y9QCZF547/examples_and_tests/examples_x64/index.html]

win-builder.r-project.org - /875Y9QCZF547/examples\_and\_tests/examples\_x64/

# win-builder.r-project.org - /875Y9QCZF547/examples\_and\_tests/examples\_x64/

---

```
[To Parent Directory]  
  
16.04.2014    14:47        81183 cached-profile.txt  
16.04.2014    14:47       530884 cached-timeseries.txt  
16.04.2014    14:47         3611 dccd-Ex.pdf  
16.04.2014    14:47           26 dccd-Ex.timings
```

---
