# Supplementary material for: Cell cycle population effects in perturbation studies
Source: Mol Syst Biol. 2014 Jul 1;10(6):732. doi: 10.15252/msb.20145172 (PMC4265054; doi:10.15252/msb.20145172)
Supplement: Supplementary file 16 — Supplementary R Packages [file msb0010-0732-sd16.zip › code/Rpackages/dccd_1.1.1.tar.gz!/dccd/test/win-builder.r-project.org/875Y9QCZF547/examples_and_tests/index.html]

win-builder.r-project.org - /875Y9QCZF547/examples\_and\_tests/

# win-builder.r-project.org - /875Y9QCZF547/examples\_and\_tests/

---

```
[To Parent Directory]  
  
16.04.2014    14:47         5622 dccd-Ex_i386.Rout  
16.04.2014    14:47         5623 dccd-Ex_x64.Rout  
16.04.2014    14:47        <dir> examples_i386  
16.04.2014    14:47        <dir> examples_x64
```

---
