# Supplementary material for: Cell cycle population effects in perturbation studies
Source: Mol Syst Biol. 2014 Jul 1;10(6):732. doi: 10.15252/msb.20145172 (PMC4265054; doi:10.15252/msb.20145172)
Supplement: Supplementary file 16 — Supplementary R Packages [file msb0010-0732-sd16.zip › code/Rpackages/dccd_1.1.1.tar.gz!/dccd/test/win-builder.r-project.org/index.html]

win-builder.R-project.org


# Building and checking R source packages for Windows.

#### This site provides services for building and checking R source packages for Windows.

This service is intended for useRs who do not have Windows
available for checking and building Windows binary packages.
Windows useRs can easily build and check their packages on Windows with the setup
described in the R Installation and Administration manual.

Please do not upload packages of other maintainers
(particularly not without changing the Maintainer field to your own e-mail address, if you have permissions to do that),
because the maintainer indicated in the maintainer field of the DESCRIPTION file get response from us.
Please do not upload BioConductor packages or CRAN packages.
Both BioConductor and CRAN do have build systems. If BioConductor or CRAN packages are not available for Windows,
there is a certainly a reason and also this service won't be able to build the package properly.

A Windows Server 2008 (64-bit) machine (2x Intel Xeon E5-2670, 8 cores each, 2.6 GHz, 32Gb RAM)
builds and checks your source packages and makes the installation log, check log and the Windows binary available.

Currently, the following R versions are running and ready for uploads under these directories:

- R-release:  R-release, currently R-3.1.0
- R-devel:  R-devel, to be R-3.2.0

#### The procedure is as follows:

1. Prepare your source package
   (using R CMD build)
   and add yourself including your e-mail address in the
   "Maintainer" field of the
   DESCRIPTION file
   (this is important,
   or you won't get any response from this server).
2. Check the package on your platform
   (using R CMD check).
3. If the package passes the checks under your platform,
   upload it using
    passive
   ftp  in binary mode
   (login as anonymous) to the directories mentioned above on
   ftp://win-builder.r-project.org/.
   Passive ftp can be performed with recent ftp clients (but not the Windows internal one)
   and using Browsers (such as Mozilla-alikes and IE) for the upload.  
   As an alternative, you can try out the upload page.
4. Wait for half an hour or so and
   check the e-mail address you have specified in the
   "Maintainer"
   field of the package's DESCRIPTION file.
   An e-mail should have arrived that will explain where the binary and the log
   files can be downloaded from: A randomly generated directory on this
   server which will be deleted after roughly 72 hours.

If your package has dependencies:

- All CRAN packages, BioConductor `software' packages, and a selection of Omegahat packages are already installed.
- Additionally it is possible to install packages serially yourself by uploading them serially:  
  The first package to be uploaded should be the one that is needed by any other packages you upload.
  Packages you installed yourself are deleted on a regular basis.
- The tools and software provided by Professor Ripley (http://www.stats.ox.ac.uk/pub/Rtools/R215x.html)
  as well as third party software products mentioned at http://cran.r-project.org/bin/windows/contrib/ThirdPartySoftware.html
  are available.

#### Disclaimer:

- You are using this service on your own risk!
- We do not guarantee privacy nor confidentiality
  of the uploaded files or data in any way:
  Names of uploaded packages are always listable by everybody.
  After the compilation process we try to hide the binary package as well as the logs
  using randomly chosen directory names.
  Nevertheless, everybody who knows or guesses those names can download those files.
- Although virus checking is performed on the compiled binary packages,
  we do not guarantee that files made available for download
  are free of malware (such as viruses or trojans).
- The results of R CMD check
  are returned from a particular given system with a particular setup and might not be reproducible on other systems.
  Hence we do not guarantee for the results of
  R CMD check.

Uwe Ligges
  
(CRAN maintainer for Windows binary packages)
  
  
This machine is hosted by the Department of Statistics, TU Dortmund, Germany.
  
  
last change: April 10, 2014
