# Supplementary material for: Cell cycle population effects in perturbation studies
Source: Mol Syst Biol. 2014 Jul 1;10(6):732. doi: 10.15252/msb.20145172 (PMC4265054; doi:10.15252/msb.20145172)
Supplement: Supplementary file 16 — Supplementary R Packages [file msb0010-0732-sd16.zip › code/Rpackages/dccd_1.1.1.tar.gz!/dccd/test/win-builder.r-project.org/upload.aspx]

win-builder upload page

**Before uploading anything, please read the the instructions and the disclaimer
at the main page: http://win-builder.r-project.org/.**


---


## R-release

For uploading R source packages to be built and checked with R-release, please use this first form:


---

## R-devel

For uploading R source packages to be built and checked with R-devel, please use this second form:


---

  
This machine is hosted by the Department of Statistics, TU Dortmund, Germany.
  
  
last change: May 20, 2012
